# Current News

**Published:** 1894-01

**Authors:** 


### Current News.

### DR. JESSE C. GREEN HONORED.

In commemoration of his fiftieth anniversary in the practice of dentistry, a banquet was tendered to Dr. Jesse Cope Green at the Union League of Philadelphia, November 4, 1893.

After the dinner, Dr. Alonzo Boice, the toast-master, and president of the Philadelphia County Dental Society, spoke of the part the Society had taken in bringing together so many representative members of the profession to pay tribute to the honored friend and guest. In introducing the first speaker of the evening, Dr. Boice said, "I notice a brother who has practised not fifty years, but forty-two years; every one in this company knows him personally. He has been identified with all the dental societies throughout the United States. He has been, and is, an original investigator, author, and teacher, placing him in the front ranks of literature and science. I refer to Professor C. N. Peirce."

Dr. Peirce spoke in part as follows:

"Mr. Chairman and Friends,—I esteem it a great privilege that I should have been invited to speak to you this evening of our honored guest, one who has for a half-century served the public, giving position and prominence to his profession, to our profession, and made a place for himself in the hearts of his associates and friends—a position which renders his advancing years not irksome, but delightful, giving weight to his discourses, and so personifies integrity that in the richness of his life the man stands before you without a shadow to obscure the inmost throbbings of his heart.

"The consciousness of a life well spent and the recollections of virtuous actions, he will tell you, far exceed stocks and bonds as a paying investment at any age. Nobility and dignity such as his, refined by courtesy, cannot be changed by increasing years.

"It matters not whether the position our beloved friend has filled and is filling be civil, professional, or domestic, the duties performed are for the benefit of the patient, the people, or the family; self has always been subordinated to the interest of those being served. You ask me why our friend is so happy and so young, yet so long in life? I answer, he does not climb the mast or run up and down the decks as do the boys, but at the helm he sits and, holding it firmly, guides the ship clear of the rocks and in the current holds her steady, doing not those things that the young do, but greater, better things.

"Let us now for a moment lift the veil and read a little from our honored friend's private record; in doing so we shall see that he was wise in recognizing that the skill to do comes of doing, and that knowledge comes by opportunities embraced, with eyes always open and, working, willing hands ever ready when and where they could be helpful."

The speaker here reviewed the long and valuable services of Dr. Green not only in his profession, but in scientific and literary lines. He spoke, too, of the doctor's friendship with such men as Wendell Phillips and George William Curtis, with Whittier, Emerson, Lowell, and Longfellow, and in closing addressed the guest as follows:

"This evening, my dear friend, our honored guest, we are all here to greet you, give you our hearts untouched by jealousy, our souls unmoved by rivalry. We cry out to you with one voice, God bless you and be with you on your happy way."

The Chairman next introduced Dr. Green as "Our Honored Guest." The doctor in responding spoke as follows:

"Friends, as I call you all in my own language, I want you all to accept my most sincere and grateful thanks for what I have experienced and felt this evening, and while I do not expect to make a speech, as our friend, I thank him for all he has said, but still feel that his picture has been overdrawn.

"I am glad to see around this table many friends whom I have long known, and to experienee the sociability we feel here to-night. I commenced the practice of dentistry when there was no such thing." The doctor then dwelt at some length upon the condition and position of dentistry a half-century ago, and of its evolution up to the present time. He spoke particularly of the advances made in dental education, of the remarkable strides in dental journalism, of the introduction of anæsthetics, etc., and closed with these modest and characteristic remarks: "When first spoken to concerning this banquet, I said it was not my nature to take a prominent place, but the question arose in my mind, Can I refuse it if by so doing it would be of use to some one or add to the pleasure of others? The great aim of life should be to benefit the people by our existence. In closing, I can certainly say that at the present time I am enjoying a 'Green' old age."

The toast-master then called upon Professor E. T. Darby, who in response said,—

"I always think of Dr. Green as about my own age, though I believe he began the practice of dentistry before I was born. I think of him as I think of Oliver Wendell Holmes, who once said that he was seventy-five years young, not seventy-five years old.

"It was twenty-eight years ago last June since I first met Dr. Green. He has from that day to this been my friend. There is no man in the profession I respect more. I have been in his shop many times, and it is one of the most interesting of places. Some of the most pleasant and instructive conversations I have ever had have been in that den of his. I look back upon our friendship as one of my most pleasant recollections. Wherever the dental profession is known, wherever Dr. Green's name is known, he is loved and respected, honored by all."

The Hon. J. Russell Young, President of the League, then made an appropriate and pleasing address. He expressed himself as being happy to join in doing honor to the modest, useful, and meritorious life of the venerable guest. He spoke further of the many noted people who had been entertained at the League, but none, he said, were more thoroughly welcome than Dr. Jesse Green.

Other toasts were responded to by Professors Wilbur F. Litch and Henry Leffmann, and Drs. E. K. Sanger, President of the Central Dental Association of New Jersey, F. L. Bassett, President of the Pennsylvania State Dental Society, E. M. Beesley, President of the New Jersey State Dental Society, L. A. Faught, of the Pennsylvania State Examining Board, and others.

Numerous letters and telegrams were also received, one from far-off California, sending congratulations and good cheer to Dr. Green.

